# Knockdown of the prognostic cancer stem cell marker Musashi-1 decreases radio-resistance while enhancing apoptosis in hormone receptor-positive breast cancer cells via p21^WAF1/CIP1^

**DOI:** 10.1007/s00432-021-03743-y

**Published:** 2021-07-22

**Authors:** Fabian M. Troschel, Heike Palenta, Katrin Borrmann, Kristin Heshe, San Hue Hua, George W. Yip, Ludwig Kiesel, Hans Theodor Eich, Martin Götte, Burkhard Greve

**Affiliations:** 1 Department of Radiation Oncology, University Hospital Münster, 48149 Münster, Germany; 2 Department of Gynecology and Obstetrics, University Hospital Münster, 48149 Münster, Germany; 3 Department of Anatomy, Yong Loo Lin School of Medicine, National University of Singapore, Singapore, 117594 Singapore

**Keywords:** RNA-binding proteins, Musashi-1, Breast cancer, Radio-resistance, p21, Apoptosis

## Abstract

**Purpose:**

While the stem cell marker Musashi-1 (MSI-1) has been identified as a key player in a wide array of malignancies, few findings exist on its prognostic relevance and relevance for cancer cell death and therapy resistance in breast cancer.

**Methods:**

First, we determined prognostic relevance of MSI-1 in database analyses regarding multiple survival outcomes. To substantiate findings, MSI-1 was artificially downregulated in MCF-7 breast cancer cells and implications for cancer stem cell markers, cell apoptosis and apoptosis regulator p21, proliferation and radiation response were analyzed via flow cytometry and colony formation. Radiation-induced p21 expression changes were investigated using a dataset containing patient samples obtained before and after irradiation and own in vitro experiments.

**Results:**

MSI-1 is a negative prognostic marker for disease-free and distant metastasis-free survival in breast cancer and tends to negatively influence overall survival. MSI-1 knockdown downregulated stem cell gene expression and proliferation, but increased p21 levels and apoptosis. Similar to the MSI-1 knockdown effect, p21 expression was strongly increased after irradiation and was expressed at even higher levels in MSI-1 knockdown cells after irradiation. Finally, combined use of MSI-1 silencing and irradiation reduced cancer cell survival.

**Conclusion:**

MSI-1 is a prognostic marker in breast cancer. MSI-1 silencing downregulates proliferation while increasing apoptosis. The anti-proliferation mediator p21 was upregulated independently after both MSI-1 knockdown and irradiation and even more after both treatments combined, suggesting synergistic potential. Radio-sensitization effects after combining radiation and MSI-1 knockdown underline the potential of MSI-1 as a therapeutic target.

**Supplementary Information:**

The online version contains supplementary material available at 10.1007/s00432-021-03743-y.

## Introduction

Breast cancer remains the most common malignancy in women worldwide, thus carrying a significant burden of morbidity and mortality (Bray et al. 2018). Individual prognosis is mainly determined by the tumor’s metastatic ability and therapy resistance (Sledge et al. 2014). Breast cancer stem cells (BCSCs) have been identified as key drivers of both features (McDermott and Wicha 2010). BCSCs constitute a cancer cell subpopulation of highly tumorigenic, chemo- and radio-resistant cells. They are known to exhibit several markers, including CD133, CD44 and aldehyde dehydrogenase (ALDH) (McDermott and Wicha 2010). Multiple pathways have been identified that contribute to stem cell maintenance. Among these, the notch pathway (including the notch protein family) has been linked to recurrence and metastasis of cancer (Meisel et al. 2020). Given their outsized importance for tumor initiation and progression, targeting BCSCs remains a key priority (McDermott and Wicha 2010).

Musashi RNA-binding proteins are one of the major players in maintaining breast cancer stem cell properties and consist of two isoforms, both with similar mRNA binding properties (Okano et al. 2005). Musashi-1 (MSI-1) is a small intracellular protein acting as a post-transcriptional gene expression regulator (Fox et al. 2015). It has been identified as a cancer stem cell marker in a variety of cancers (Fox et al. 2015). In breast cancer, it is known to support tumor growth (Wang et al. 2010) as well as stem-like capacities (Lagadec et al. 2014), mainly by upregulating the notch pathway (Wang et al. 2010; Lagadec et al. 2014). It has also been linked to decreased levels of p21^WAF1/CIP1^ (Wang et al. 2010), a cyclin-dependent kinase inhibitor and, as such, a key versatile cell cycle protein that tends to inhibit breast cancer cell growth (Kreis et al. 2019). In a previous study limited to triple-negative breast cancer (TNBC), our group first linked MSI-1 to cell death mediation, key to therapeutic success in breast cancer (Troschel et al. 2020). In this study, we set out to understand the prognostic significance of MSI-1 expression in breast cancer and subsequently investigated ramifications of targeting MSI-1 for apoptosis and therapy resistance in hormone receptor-positive breast cancer cells.

## Materials and methods

### Cell culture & siRNA transfection

The hormone receptor-positive, luminal breast cancer cell line MCF-7 derived from a 69-year-old patient in 1970 (Soule et al. 1973) was acquired from American Type Culture Collection (ATCC)/LGC Standards (Wesel, Germany). Cell line authenticity was confirmed via short tandem repeat (STR) analysis. Cells were cultured as previously described (Troschel et al. 2018). Culture conditions were 37 °C with 5% CO_2_. Cells were cultured in RPMI 1640 medium (PAN Biotech, Aidenbach, Germany). 1% penicillin/streptomycin and 10% FCS (both Thermo Fisher Scientific, Waltham, MA, USA) were added.

For transient transfection, two MSI-1 siRNAs were used in equal concentrations (Applied Biosystems, Thermo Fisher Scientific, sequences in Supplementary Table S1). siRNAs were concentrated to 20 nM combined. Then, 4 µl siRNA, 4 µl Dharmafect transfection medium (Dharmacon, Lafayett, CO, USA) and 152 µl Opti-MEM (Gibco, Thermo Fisher Scientific) were added to each well in a 6-well plate. Cells were incubated for 24 h. Then, medium was replaced, and cells were cultured for another 48 h before subsequent experiments were started.

### Quantitative polymerase chain reaction (qPCR)

The RNeasy Mini Kit was used to isolate mRNA after transfection according to manufacturer’s instructions. Reverse transcription was performed with the High-Capacity cDNA Reverse Transcription Kit (all Qiagen, Venlo, The Netherlands). Finally, a Rotor-Gene Q machine was used for the chain reaction. Taq Man probes (all Applied Biosystems, Foster City, CA, USA) normalizing to 18S expression were used (Supplementary Table S2). RNA quality was determined via biophotometer (Eppendorf, Hamburg, Germany) with A260/280 rates between 1.8 and 2.0 considered appropriate, as before (Troschel et al. 2018). Data are expressed as fold changes using the 2^-∆∆Ct^ method comparing MSI-1 knockdown cells to controls.

### Western Blot

Western Blot experiments were performed as previously described (Troschel et al. 2020). About 10^7^ cells were trypsinized and lysed. Subsequently, 30 µg of whole protein was electrophoresed and transferred to nitrocellulose and thus used for immuno-detection. Antibody binding was visualized using ECL peroxidase blotting substrate (Thermo Fisher Scientific). Then, quantification of luminescence was performed with a Fusion SL System (Peqlab, Erlangen, Germany). Primary and secondary antibody details are shown in Supplementary Table S3.

### Mammosphere formation

Equal numbers of control and MSI-1 knockdown cells were seeded into coated six-well plates (Greiner, Kremsmünster, Austria) 24 h after siRNA transfection. To generate sphere formation, Spheromax CSC Medium (Promocell, Heidelberg, Germany) was added. After 7 days, sphere formation was assessed macroscopically. Additionally, sphere area in μm^2^ was quantified microscopically. Here, we photographically compared four microscopic fields for controls and knockdown cells. For area quantification, we used a previously published ImageJ Macro (Ivanov et al. 2014).

### Apoptosis measurements

Subsequent to washing with phosphate-buffered serum (PBS), cells underwent the Annexin V/propidium iodide (PI) assay (Thermo Fisher Scientific), as detailed by the manufacturer and as previously described (Greve et al. 2009). Measurement was performed on a flow cytometer (CyFlow space, Sysmex/Partec, Münster, Germany) using FloMax software (Quantum Analysis, Münster, Germany) to visualize and manage flow data. For interpretation, the fourth quartile in the measurement graph indicated apoptotic cells as cells were positive for Annexin V (binding to phosphatidylserine as cell membranes lose lipid asymmetry during apoptosis), but negative for propidium iodide, showing cells were apoptotic but cell membranes remained intact (Crowley et al. 2016).

### Colony formation and radio-resistance

After transfection as described above, predefined equal numbers of cells were mixed with Matrigel (MethoCult Express, StemCell, Vancouver, Canada) and seeded in cell culture dishes (Nunc, Langenselbold, Germany). After 10 days of incubation, number of cell colonies was determined. Colonies were defined as contiguous cell groups (clones) of more than 50 cells. The number of colonies in MSI-1 knockdown cell dishes was calculated relative to the number of colonies in control cell dishes to understand changes in cell proliferation patterns.

Clonogenic assays were also used for determination of radio-resistance. Here, cells were treated with 6 Gy of radiation from a clinical TrueBeam linear accelerator (Varian, Palo Alto, CA, USA). The dose was chosen to mirror 5 Gy doses from the GSE59732 dataset. After 10 days, colonies were counted as described above. Plating efficacy (PEf) was calculated as PEf = number of colonies/number of seeded cells. Surviving fractions (SF) were then determined as SF = PEf (irradiated)/PEf (control).

### MTT (3-(4,5-dimethyl-2-yl)-2,5-diphenyltetrazolium bromide) cell viability assay

MTT assays were performed as previously described (El-Nadi et al. 2020). Briefly, 4000 cells/well of MSI-1 knockdown and control cells were seeded in 96-well plates 48 h after transfection and cultured for an additional 24 h. Then, chemotherapy was added: specifically, varying doses of cisplatin, and doxorubicin were used. MTT reagent was added 72 h later and measurements were performed after 24 h at 570 nm.

### Database gene expression analyses

For survival analyses, we used all DNA microarray data available in the bc-GenExMiner (Jézéquel et al. 2012). To avoid overfitting and false-positive results, we used default settings (median MSI-1 expression as cutoff, log-rank analyses) for three outcomes of disease-free distant metastasis-free and overall survival. In a secondary analysis, we investigated ER-positive breast cancers only.

For gene expression correlation analyses, we used all DNA microarray breast cancer samples available (Jézéquel et al. 2013). Again, we used default settings to avoid false detection rates due to multiple testing. The correlation tool provides Pearson correlations, including Pearson’s *r* and the respective *p* value. These are the results presented in Table 1.

**Table 1:** Correlation of gene expression based on DNA microarray data from the bc-GenExMiner tool

MSI1 correlations	Pearson’s *r*	*p* value	Patient *n*
Notch-1	0.11	*p* < 0.001	*n* = 9417
Notch-3	0.05	*p* < 0.001	*n* = 9650
Nanog	0.07	*p* < 0.001	*n* = 8776
Tdgf1	0.06	*p* < 0.001	*n* = 4733
Nestin	0.11	*p* < 0.001	*n* = 9095
P21 (CDKN1A)	− 0.03	*p* = 0.008	*n* = 9650

Pearson’s correlations were used and Pearson’s *r*, respective *p* value and number of measurements used are given. For more details, please refer to “Methods” section

Finally, we correlated MSI-1 expression and the Nottingham Prognostic Index (NPI) using the same database. Again, all available breast cancer cases were included (the database does not allow sub-stratification, e.g., by estrogen receptor positivity). For expression comparison, the Dunnett–Tukey-Kramer test was performed by the tool.

### ROC plotter

To better understand associations between MSI-1 and chemotherapy response, we used the ROC plotter (Fekete and Győrffy 2019). Here, we checked all included breast cancer patients receiving “any chemotherapy” as the primary analysis with a secondary analysis for estrogen receptor-positive patients only. All other settings were the pre-programmed default settings. As results, the website then reported expression levels of MSI-1 with differences assessed with the Mann–Whitney *U* test. Similarly, an AUC analysis was performed for the strongest cutoff with *p* value and AUC statistic presented.

### GSE59732 and GSE59733 GEO dataset analyses

For pre- and post-irradiation comparisons, we utilized the connected GSE59732 and GSE59733 databases. Here, gene expression profiling in response to radiation treatment is presented in 9 paired breast cancer patient samples (database GSE59733) as well as in cell lines (database GSE59732). We used all paired primary patient samples and the MCF-7 cell line data. Data are presented as log2 RMA signal.

### Statistics

All experiments were performed independently at least three times in duplicates. Student’s *t* test was used to assess differences. Level of significance was defined as *p* < 0.05. Fold changes are visualized as mean ± standard error of the mean (s.e.m.). For primary samples, paired *t* test was used. Pearson correlation was used for expression correlation in the bc-GenExMiner database with *r* and *p* values given.

## Results

### MSI-1 is a negative prognostic marker for multiple outcomes in breast cancer patients

**Fig. 1:**
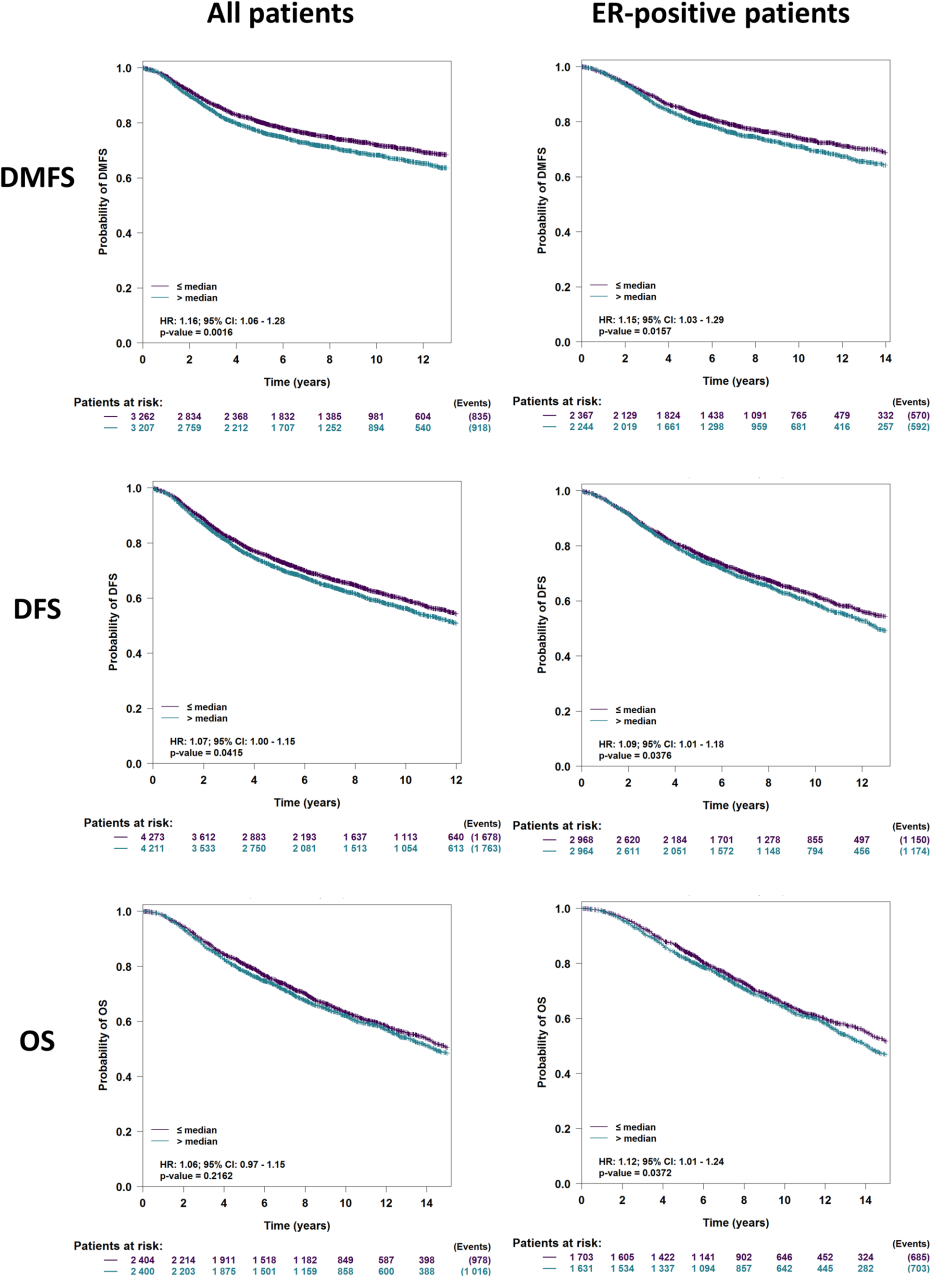
MSI-1 is associated with survival outcomes in breast cancer patients. Across all breast cancer patients (left column), higher than median MSI-1 expression results in decreased distant metastasis-free survival (DMFS, hazard ratio [HR] = 1.16, *p* = 0.0016) and disease-free survival (DFS, HR = 1.07, *p* = 0.042) and tends to decrease overall survival (OS, HR = 1.06, *p* = 0.22). Similarly, in the estrogen receptor (ER)-positive group (right column), greater than median MSI-1 expression is associated with decreased DMFS (HR = 1.15, *p* = 0.016), DFS (HR = 1.09, *p* = 0.038) and OS (HR = 1.12, *p* = 0.038). Analyses (visualization and log-rank tests) were performed using the bc-GenExMiner tool (Jézéquel et al. 2012)

A previous study linked Musashi expression to overall survival (OS) in a small cohort of breast cancer patients (Wang et al. 2010). Here, we leveraged a large database of gene expression studies to characterize the association between MSI-1 and multiple outcomes including OS, disease-free survival (DFS), and distant metastasis-free survival (DMFS). We performed analyses both in all breast cancer samples and in hormone receptor-positive breast cancers only. Using the bc-GenExMiner tool (Jézéquel et al. 2012), we were able to identify MSI-1 as a negative prognostic marker for DFS and DMFS for all breast cancer patients and estrogen receptor-positive breast cancer patients only (*p* < 0.05 in all cases, Fig. 1). Overall survival tended to be negatively associated with MSI-1 but was significantly associated with MSI-1 in estrogen receptor-positive patients only.

These findings informed our following studies to target MSI-1.

### MSI-1 knockdown results in downregulation of key stem cell markers, potentially rendering breast cancer cells vulnerable to anti-cancer signaling

Similar to previous findings (Troschel et al. 2020), siRNA-based MSI-1 knockdown downregulated MSI-1 expression to a level of roughly 15% when compared to controls (*p* < 0.001). In western blot analyses, MSI-1 was downregulated as well, albeit less strongly (*p* < 0.05, Supplementary Figure S1).

We first reconfirmed that MSI-1 knockdown downregulated breast cancer stem cell characteristics: Mammosphere formation was significantly repressed following MSI-1 knockdown, both in number and in size, confirming previous findings (Supplementary Figure S2) (Wang et al. 2010). We then proceeded to investigate stem cell-related genes: given well-known associations with the notch pathway (Wang et al. 2010) we quantified notch-1 and notch-3, both of which were significantly downregulated by roughly 40% subsequent to MSI-1 silencing (*p* < 0.05, Supplementary Figure S3). Expression of Tdgf1, a stem cell factor related to notch signaling sensitivity (Watanabe et al. 2009), but not previously reported as a MSI-1 regulatory target, was similarly decreased (*p* < 0.05).

Nanog, a transcription factor key to cellular reprogramming and a known breast cancer stem cell marker (Harati et al. 2019), was also expressed at lower levels (*p* < 0.05).

Finally, Nestin, a stem cell intermediate filament (Asleh et al. 2018), was expressed less in MSI-1 knockdown cells, barely missing the level of significance (*p* = 0.052).

We then confirmed these correlations using the bc-GenExMiner tool (Table 1). In close to 10,000 breast cancer samples (using default settings to avoid multiple testing, thus including a heterogeneous cohort of all breast cancer patients available), notch-1 and notch-3 expression was positively associated with MSI-1. Similar trends were observed for Nanog and Tdgf1. In this analysis, Nestin was positively correlated with MSI1 expression, meeting the level of statistical significance. However, given the heterogeneity of the data (multiple datasets were pooled, different breast cancer types included and expression of the markers varied widely), Pearson’s *r* values were very low despite strong statistical significances.

These findings, including the identification of new additional stem cell markers attenuated after MSI-1 knockdown led us to hypothesize that breast cancer cells may be vulnerable to anti-proliferative and pro-apoptotic signaling.

### MSI-1 downregulation increases p21 expression in MCF-7 cells, resulting in increased apoptosis and decreased proliferation

**Fig. 2:**
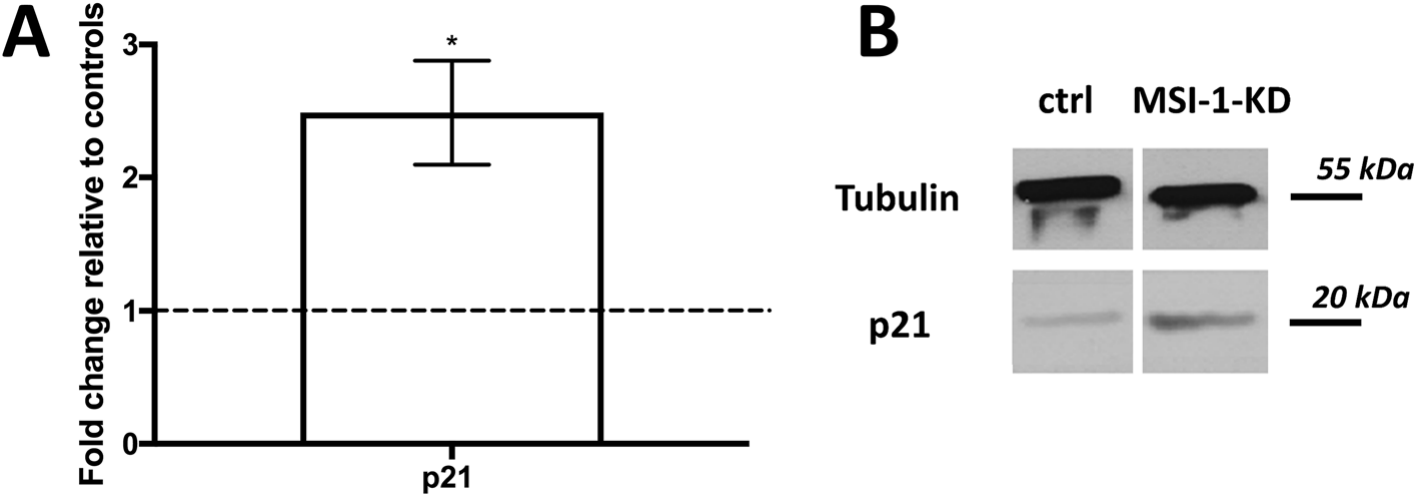
Expression of p21 in MCF-7 cells after MSI-1 knockdown, compared to controls. Cells were transfected with a control siRNA and MSI-1 siRNA, respectively. Then, western blot measurements and quantification were performed as detailed in the Methods section (*n* = 3, *p < 0.05, error bars indicate s.e.m.). Overall results are shown in panel **A**; representative measurements are shown in panel **B**

Besides notch pathway constituents and additional stem cell markers, the cell cycle protein p21 has been described as a regulatory target of MSI-1 (Battelli et al. 2006; Wang et al. 2010; Jadhav et al. 2016). Correlation analysis using the bc-GenExMiner database revealed a negative association between MSI-1 and p21 in breast cancer specimens (Table 1). For functional analyses, we next chose an MSI-1 siRNA knockdown approach in the ER-positive cell line MCF-7. In accordance with previous data (Wang et al. 2010), we observed a significantly stronger p21 expression in western blot investigations, more than doubling the control siRNA-transfected levels after MSI-1 knockdown in our experimental system (Fig. 2).

**Fig. 3:**
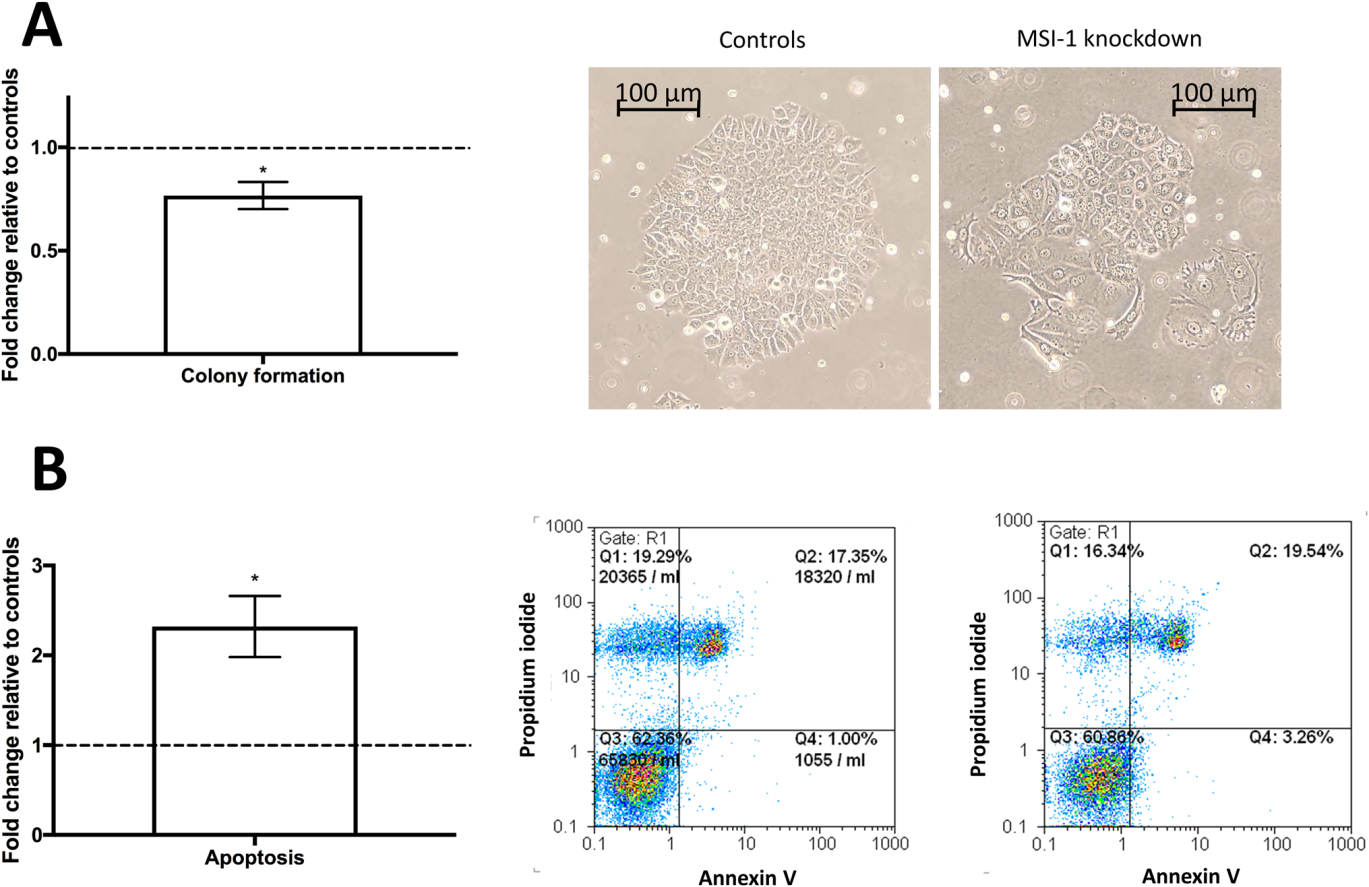
Colony formation and apoptosis in MCF-7 cells after MSI-1 knockdown, compared to controls. Cells were transfected with a control siRNA and MSI-1 siRNA, respectively. **A** Colony formation is reduced after MSI-1 knockdown, a representative colony for control si-RNA transfected cells (middle) and MSI-1 knockdown cells (right) are also visualized. **B** Apoptosis is upregulated subsequent to MSI-1 silencing with representative images for controls (middle) and MSI-1 silenced cells (right). Apoptotic cells can be found in the fourth quartile (Q4, bottom right). Measurements were performed as detailed in the Methods section (*n* = 3, *p < 0.05, error bars indicate s.e.m.)

Given the decisive role of p21 in the regulation of breast cancer cell proliferation (Li et al. 2020), we confirmed that colony formation was significantly downregulated after MSI-1 knockdown: clonogenic capacity was down by 25% (Fig. 3A).

However, p21 has also been described to be a regulator of apoptosis and cell damage response (Weiss 2003). Thus, we also investigated apoptosis rates and found that MSI-1 downregulated cells were more than twice as likely to be apoptotic when compared with control siRNA-transfected cells (Fig. 3B).

### Irradiation increases p21 levels in MCF-7 and primary patient data

**Fig. 4:**
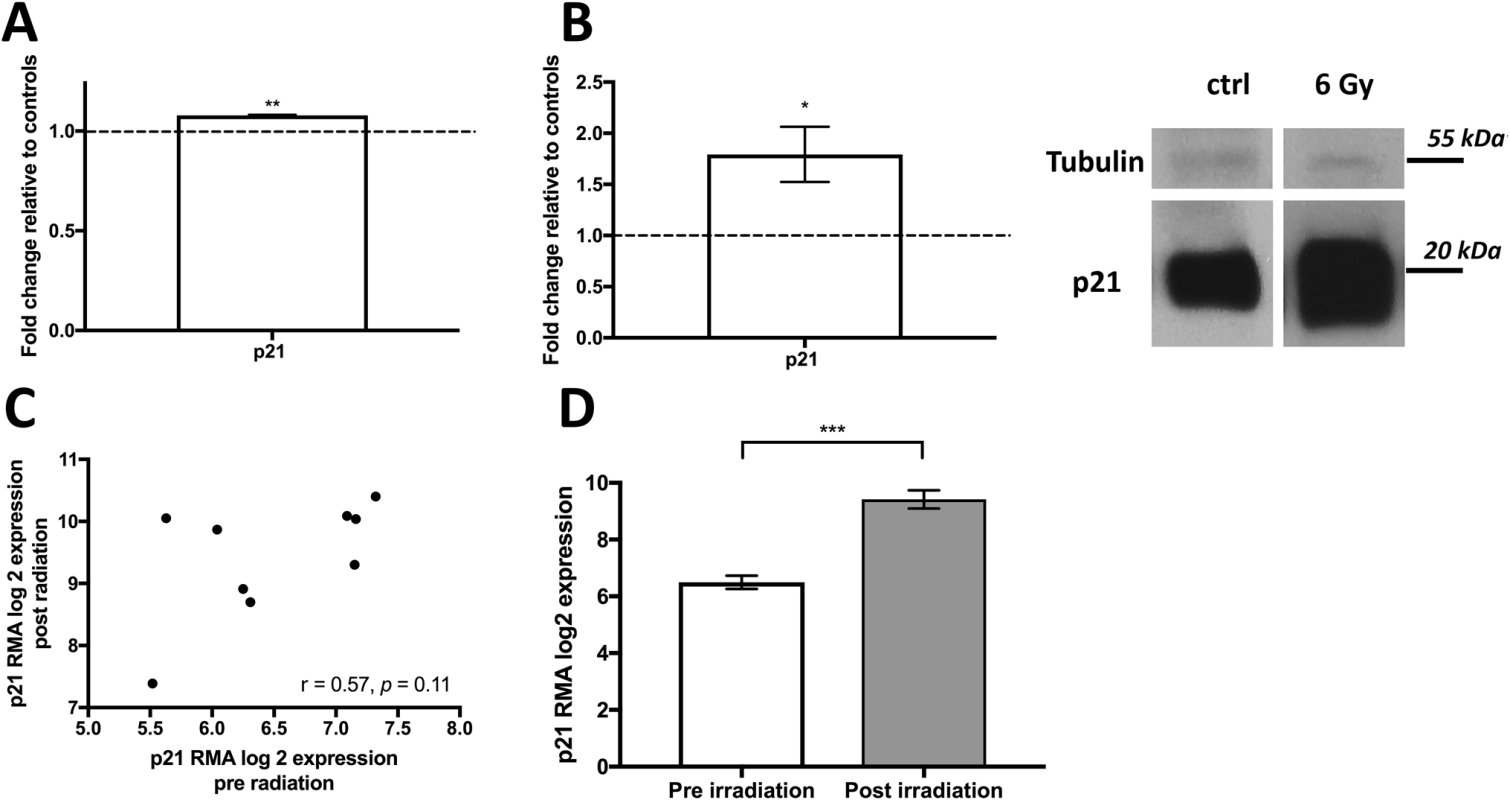
Expression of p21 after irradiation. **A** p21 mRNA levels in MCF-7 cells after 5 Gy of irradiation as analyzed based on the GSE59732 dataset. **B** p21 protein levels in MCF-7 cells after 6 Gy vs. after no irradiation in our cell line experiment (left) with representative images (right) (*n* = 3, *p < 0.05, **p < 0.01, error bars indicate s.e.m.). Please note that tubulin and p21 were stained on the same gel simultaneously. As p21 was highly expressed gel exposure times were shortened, thus limiting tubulin staining. **C** Expression levels of p21 in 9 patient breast cancer samples pre- and post-irradiation as analyzed based on the GSE59733 dataset. Pearson’s correlation analysis was performed. **D** Mean expression of p21 pre- and post-irradiation. A paired *t* test was performed (***p < 0.001, error bars indicate s.e.m.). Data were normalized using Robust Multichip Average (RMA)

After finding that MSI-1 knockdown increased p21 expression, we independently aimed to also understand irradiation-induced effects on p21. We thus compared expression of p21 after 5 Gy irradiation to cells with no irradiation in the MCF-7 cell line, utilizing the GSE59732 GEO database. P21 gene expression was significantly upregulated after irradiation (Fig. 4A). We were able to confirm the data using MCF-7 western blot experiments of our own, after a similar dose of 6 Gy (Fig. 4B).

We also utilized the connected GEO database GSE59733 of 9 patient breast cancer samples prior and subsequent to irradiation to compare p21 expression levels. There was a good correlation between pre- and post-radiation levels in the paired samples (Fig. 4C, Pearson’s *r* = 0.55, *p* = 0.11), indicating that p21 levels reacted to irradiation consistently across all samples. In paired analyses, there was a strong (*p* < 0.001) increase in p21 levels after irradiation (Fig. 4D).

### MSI knockdown prior to irradiation leads to radiosensitization in MCF-7

**Fig. 5:**
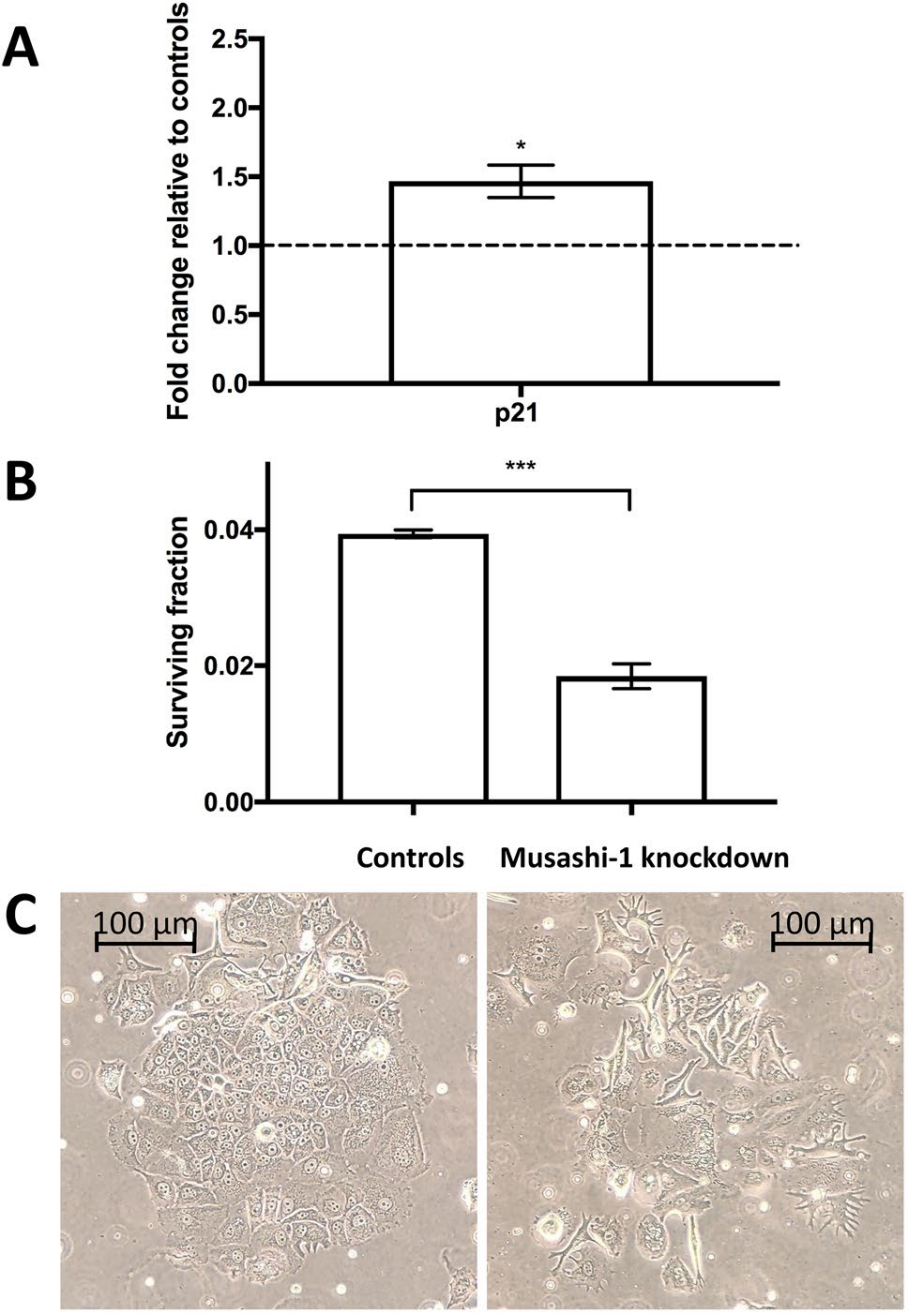
Clonogenic survival after irradiation. **A** p21 is stronger expressed in MSI-1 knockdown cells after 6 Gy irradiation compared to control siRNA-transfected cells after 6 Gy. **B** Cells undergoing MSI-1-knockdown exhibited a strongly decreased clonogenic ability when compared to controls (*p < 0.05, ***p < 0.001, error bars indicate s.e.m.). **C** Representative images of a colony of control cells (left) and numerous, non-contiguous cells after MSI-1 transfection (right), both after a radiation dose of 6 Gy

p21 has been described as a radio-sensitizer in breast cancer (Yang et al. 2012), as has notch pathway downregulation (Yahyanejad et al. 2016). Thus, we hypothesized that MSI-1 knockdown may increase radio-sensitization. First, we tested whether MSI-1 knockdown and irradiation had synergistic effects on p21 expression, as both treatments had similar effects on p21 levels independently from each other. After MSI-1 knockdown and 6 Gy of irradiation, p21 levels were expressed at higher levels when compared with control siRNA-transfected cells after 6 Gy irradiation (Fig. 5, representative blots in Supplementary Figure S4).

Subsequently, we showed that clonogenic ability is strongly reduced by about 50% in MSI-1-silenced cells compared to controls after irradiation (Fig. 5). This indicates an additional, irradiation-related effect added to the previously described anti-proliferative pro-apoptotic effect in non-irradiated cells, possibly via p21 overexpression.

### Low MSI-1 expression is predictive of good chemotherapy response

**Fig. 6:**
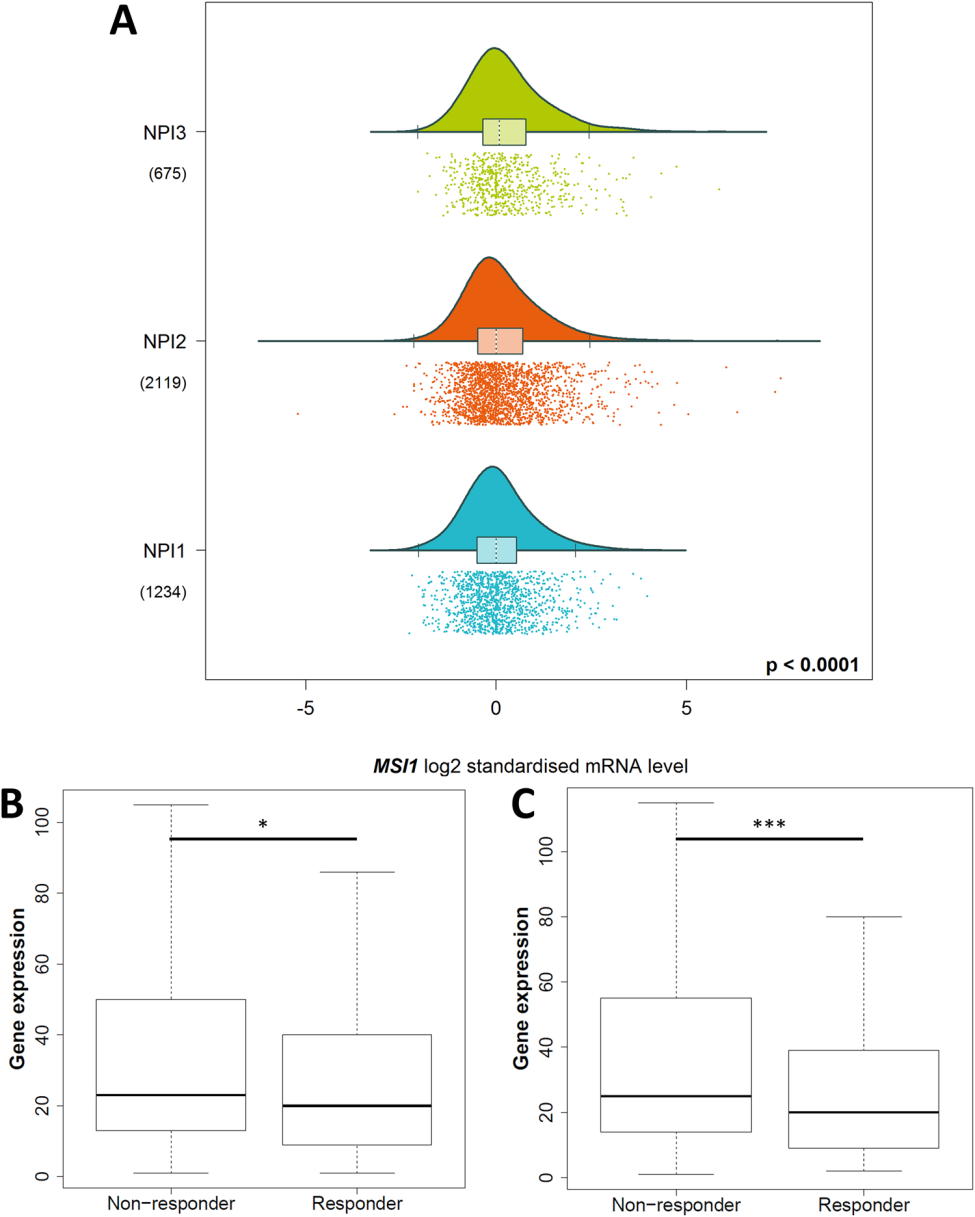
MSI-1 expression is associated with chemoresistance. **A** MSI-1 expression is highest in Nottingham Prognostic Index (NPI) grade III breast cancer samples and consecutively lower across NPI II and NPI I. This figure was generated using the bc-GenEx-Miner tool (Jézéquel et al. 2013). **B**, **C** MSI-1 expression is lower in breast cancers with complete response to chemotherapy, both in all cancer tissues (*p* = 0.016, **B**) and estrogen receptor-positive tumors only (*p* < 0.001, **C**). This figure was generated using the ROC plotter data tool (Fekete and Győrffy 2019) and slightly amended to reflect statistical significances

p21 has also been shown to be negatively correlated with chemoresistance (Hou et al. 2017; Mu et al. 2019). Thus, we hypothesized that MSI-1 knockdown may attenuate chemoresistance via p21 overexpression. Again using the bc-GenExMiner tool, we found MSI-1 to be associated with the Nottingham Prognostic Index (NPI) (Fig. 6A): here, MSI-1 expression is lowest in the least malignant NPI grade I and subsequently higher with highest expression in the most malignant NPI grade III (*p* < 0.05). The NPI is correlated with survival in breast cancer patients, underlining that MSI-1 has prognostic significance in breast cancer (Fong et al. 2015). However, the NPI is also known to be associated with chemoresistance, with NPI grade 3 associated with the highest chemoresistance (Tan et al. 2016), pointing to a relationship between MSI-1 expression and chemoresistance. Then, utilizing the ROC plotter data tool (Fekete and Győrffy 2019), we were able to directly connect MSI-1 expression and chemoresistance: using the “any chemotherapy” option (and default settings otherwise), patients with a complete response (CR) expressed significantly lower levels of MSI-1, both for all (*p* = 0.016, Fig. 6B) and for estrogen receptor-positive patients (*p* < 0.001, Fig. 6C). Similarly, the receiver operating characteristic (ROC) analyses rendered significant results (*p* < 0.001 for both groups). However, area under curve (AUC) values remained rather low (0.54 for all and 0.57 for ER-positive cases).

We subsequently followed these findings up with MTT tests in MCF-7 cells for cisplatin and doxorubicin. However, while a strong decline in viability was seen in MSI-1 knockdown cells before chemotherapy was added (Supplementary Figure S5A), no additional chemo-sensitizing effects became apparent (Supplementary figure S5B–D).

## Discussion

In the present study, we investigated the significance of MSI-1 for survival outcomes in breast cancer as well as the molecular rationale for targeting MSI-1. We found that MSI-1 silencing resulted in reduced stem cell marker expression and cell proliferation while increasing apoptosis. When submitting MSI-1 downregulated cells to irradiation, a radio-sensitizing effect was seen.

### MSI-1 is a negative prognostic factor in breast cancer

Leveraging the availability of large-scale data analysis tools, we provide an in-depth view of the negative prognostic role of MSI-1. While MSI-1 had previously been linked to overall survival in a small cohort of 140 patients (Wang et al. 2010), our findings from a large database provide a more nuanced perspective: while we were able to establish MSI-1 as a relevant factor for DFS and DMFS, we could only confirm an association with OS in hormone receptor-positive patients. In the entire cohort, there was a trend towards worse OS in MSI-1 high-expressing patients, but no significance was found (*p* = 0.2). Our findings regarding DMFS are supported by a previous study that reported that MSI-1 levels in nodal metastatic breast cancer were higher than in node-negative breast cancer (Wang et al. 2010). Besides breast cancer, MSI-1 is also prognostically relevant for other tumor entities, including colon cancer (Li et al. 2011) and glioblastoma (Vo et al. 2012).

Taken together, our analyses suggest a key role for MSI-1 for at least two of three main outcome parameters and a significant potential for MSI-1 targeting as a therapeutic approach.

### MSI-1 acts as a stem cell marker and modifier

Previous studies indicate that MSI-1 is a positive regulator of the notch pathway by directly targeting the notch repressor mnumb (Lagadec et al. 2014), thus supporting BCSC maintenance (Wang et al. 2010; Troschel et al. 2020). Our investigation reconfirms these findings as notch-1 and notch-3 were downregulated after MSI-1 silencing in MCF-7 cells. Subsequently, stem cell maintenance, as quantified by mammosphere formation (Cioce et al. 2010), was strongly repressed, similar to previous results (Wang et al. 2010). Mammosphere formation has also been correlated with tumorigenity in vivo, underlining the potential anti-tumorigenic effect subsequent to MSI-1 knockdown (Cariati et al. 2008). Additionally, Tdgf1, a factor not previously described as regulated by MSI-1 that sensitizes breast cancer cells to notch signaling (Watanabe et al. 2009), was also reduced. Notch-1, notch-3 and Tdgf1 were positively correlated with MSI-1 in the database analysis, underlining the evidence for MSI-1’s role in maintaining notch stem cell signaling. Notch-1 is a poor prognostic factor in breast cancer (Zhong et al. 2016).

Nanog is another breast cancer stem cell marker (Jeter et al. 2016) that was negatively influenced by the MSI-1 knockdown. Our findings are thus in line with other breast cancer studies (Wang et al. 2010; Lagadec et al. 2014). Interestingly, Nanog is also known as a mediator of radio-resistance in breast cancer (Harati et al. 2019), offering further incentive to investigate radiation response. Besides, Nanog expression is also correlated with clinical stage (Saravi et al. 2019), well in line with MSI-1’s prognostic relevance.

Nestin is a stem cell intermediate filament (Asleh et al. 2018). While there was only a positive trend between MSI-1 and Nestin expression in MCF-7 cells, the correlation met levels of significance in the dataset analysis. Nestin is an independent predictor of worse prognosis in breast cancer (Zhang et al. 2020).

Stem cell characteristics including notch pathway elements are associated with decreased apoptosis and increased proliferation (Suman et al. 2013). Thus, stem cell inactivation is a possible molecular mechanism behind increased apoptosis and reduced colony formation we observed after MSI-1 knockdown in this study.

### Role of p21 subsequent to MSI-1 knockdown for proliferation and apoptosis

p21 is a well-known, yet controversial signaling molecule in breast cancer (Kreis et al. 2019). It is known to be a direct translational MSI-1 target (Battelli et al. 2006), including in breast cancer (Wang et al. 2010), thus explaining the upregulation seen after MSI-1 knockdown. P21 has been attributed several key characteristics.

In breast cancer, stem cell maintenance is known to be negatively regulated by p21 (Han et al. 2016). Conversely, stem cell maintenance is enhanced if p21 is downregulated (Jain et al. 2015). This is in line with our findings which demonstrate an increase of p21 and a decrease of stem cell characteristics subsequent to MSI-1 knockdown. It constitutes another mechanistic explanation for the loss of BCSC characteristics besides the MSI-1-mediated targeting of the notch repressor mnumb discussed above.

P21 has an anti-proliferative effect in breast cancer. When p21 is injected into a mouse model, breast cancer tumor growth is significantly repressed (Ibnat et al. 2019). Similarly, upregulation of p21 reduced (Wang et al. 2010) and downregulation promoted cell proliferation (Li et al. 2020) in other studies. This is in line with our investigation which found proliferation to be strongly decreased after MSI silencing while p21 expression was increased.

While some investigations highlight an anti-apoptotic role for p21 (Fan et al. 2003), multiple studies have found that p21 has the potential to induce apoptosis if artificially upregulated (Jiang et al. 2014; Tor et al. 2015; Giordano et al. 2017). The specific role may hinge upon the cancer type or the exact intracellular localization of p21 with a nuclear localization linked to pro-apoptotic signaling (Crispi 2012; Shamloo and Usluer 2019). In this present case, p21 upregulation subsequent to MSI-1 downregulation had an anti-proliferative and pro-apoptotic effect.

Most studies have indicated that p21 is repressed in breast cancer cells compared to normal breast cells (Pellikainen et al. 2003). However, survival implications remain controversial (Zohny et al. 2019) and p21 has been described as both oncogenic and tumor-suppressive (Kreis et al. 2019). In this study, functional analyses indicate that MSI-1-dependent p21 upregulation is tumor-suppressive, similar to findings in endometrial carcinoma (Götte et al. 2011).

### MSI-1 downregulation radiosensitizes MCF-7 breast cancer cells and may lead to chemosensitization

We found that MSI-1 downregulation results in a loss of clonogenic ability of MCF-7 cancer cells after irradiation. We irradiated cells with 6 Gy to reflect the 5 Gy radiation dose from the GSE59732 database we had previously used. The small discrepancy is due to technical reasons but is highly unlikely to have changed results in a significant way.

Presently, radiosensitization is an important aim for breast cancer research and therapy (Yahyanejad et al. 2016). This study suggests MSI-1 may be an interesting potential target to that end. Two possible explanations come to mind.

First, stem cells are known to be radio-resistant, specifically if notch and nanog signaling are high (Harati et al. 2019). The decrease in notch molecules and nanog expression seen after MSI-1 downregulation may thus explain the radio-sensitizing effect.

Second, the MSI-1-mediated p21 increase may also play a role. In our study, we show a similar effect of MSI-1 knockdown and irradiation on p21 expression: both independently increase p21 levels. Subsequently, we demonstrate that both treatments combined lead to higher levels of p21 than irradiation alone, suggesting synergistic potential. In turn, p21 overexpression may reduce proliferation while increasing apoptosis, resulting in a radiosensitizing effect with reduced clonogenic cell survival. In fact, previous investigations have linked p21 expression to radiosensitization in breast cancer (Yang et al. 2012; Kim et al. 2015; Xie et al. 2016).

MSI-1-mediated modulation of radiation resistance observed in the present study is in line with previous findings in glioblastoma (Lin et al. 2018), colon cancer (Sureban et al. 2008), and our previous findings in MDA-MB-231 TNBC cells (Troschel et al. 2020). They help expand the relevance of the radiosensitizing effect and establish MSI-1 as a potential target for improved radiotherapy-based cancer cell eradication.

Additionally, our database research demonstrates compelling evidence that MSI-1 knockdown may be associated with chemosensitization as low MSI-1-expressing tumors were more likely to have a less malignant NPI grade and to show a complete response after chemotherapy. However, AUC statistics indicated prognostic value was somewhat limited. We hypothesize that this may be due to breast cancer heterogeneity and the lack of granularity in the outcome variable (differentiating complete response vs. no complete response only). Additionally, previously published links between p21 and chemoresistance showing inverse correlations demonstrate a plausible mechanistic explanation for the effects (Hou et al. 2017; Mu et al. 2019). Unfortunately, our MCF-7 cell line data did not support the findings above, so the picture is not entirely clear here. We believe that the primary patient data offer arguably more compelling evidence. In vitro, the absence of the tumor microenvironment in our MCF-7 cell culture may have acted as a confounder (and even the MCF-7 data showed a strong decrease in cell viability through MSI-1 knockdown alone). Nonetheless, additional data are needed to substantiate MSI-1-based chemosensitization effects. In any case, MSI-1 knockdown demonstrated overwhelmingly favorable outcomes in the present research and continues to be an exciting scientific research opportunity.

Finally, it is worth discussing the effect of MSI-1 knockdown on cancer stem cells versus the general cancer cell population. As mentioned above, multiple studies, including the present work, suggest that MSI-1 may be a marker of cancer stem cells and/or linked to cancer stem cell maintenance. Limiting the effects mediated by MSI-1 to cancer stem cells only, however, would be insufficient to explain the findings of this study. While cancer stem cells undoubtedly play key roles in MCF-7, their percentage relative to the general population is very limited (Engelmann et al. 2008; Cioce et al. 2010; Li et al. 2017; Xu et al. 2018). As the changes in cell characteristics quantified in our experiments oftentimes reach significant strength, this points to changes in non-stem cells also. Our flow cytometric experiments further underline the effect on non-stem cells as cell histograms demonstrate uniform changes of the entire population towards a higher likelihood of apoptotic features. Importantly, effects do not seem to be limited to a subset of cells here. We thus assume that both cancer stem and normal cancer cells are changed by the MSI-1 knockdown.

However, normal cancer cells may not be directly affected by MSI-1 loss, but rather indirectly by loss of cancer stem cells in the tumor environment. A cancer stem cell-rich microenvironment has been shown to substantially affect non-cancer stem cells (Bhat et al. 2019; López de Andrés et al. 2020). In any case, whether directly or indirectly, our findings support that MSI-1 loss affects a broad segment of cancer cells and is likely not limited to cancer stem cells.

There are several limitations to this study. First, while we rely on primary patient data for nearly all analyses, experimental proof-of-concept is performed in MCF-7 breast cancer cells only. Given the well-known heterogeneity of breast cancer, this limits applicability of the findings. Second, the database gene expression correlations only demonstrate weak strength of associations. However, this is likely tumor heterogeneity in the database, as underlined by the fact that even neighboring notch-1 and notch-2 only demonstrate a Pearson’s *r* of 0.13 in the dataset. Thus, we hypothesize that low Pearson’s *r* values should not prematurely be discounted. Third, in western blot analyses, MSI-1 expression decrease after knockdown was less pronounced when compared to qPCR analyses. This is likely due to the binding of the other MSI protein family member, MSI-2, that is nearly identical with MSI-1 (Sakakibara et al. 2001), but was not targeted for knockdown. Finally, in accordance with this study’s aim, not all associations seen via qPCR were confirmed via Western Blot, especially when not relevant to the key findings.

## Conclusion

In conclusion, MSI-1 is a prognostically relevant marker in breast cancer. Silencing MSI-1 results in downregulation of stem cell gene expression and upregulation of cell cycle and apoptosis regulator p21. Functionally, loss of MSI-1 expression leads to decreased proliferation and therapy resistance and increased apoptosis in MCF-7 cells. The present study underlines the potential of MSI-1 as a therapeutic target in breast cancer.

## Supplementary Information

Below is the link to the electronic supplementary material.

Supplementary file 1 (DOCX 2825 KB)

## Data Availability

The authors confirm that the data supporting the findings of this study are available within the article and its supplementary materials.
