# Supplementary material for: Knockdown of the prognostic cancer stem cell marker Musashi-1 decreases radio-resistance while enhancing apoptosis in hormone receptor-positive breast cancer cells via p21WAF1/CIP1
Source: J Cancer Res Clin Oncol. 2021 Jul 22;147(11):3299–312. doi: 10.1007/s00432-021-03743-y (PMC8484224; doi:10.1007/s00432-021-03743-y)
Supplement: Supplementary file 1 — (DOCX 2825 KB) [file 432_2021_3743_MOESM1_ESM.docx]

Supplementary Material

**Supplementary Table S1.** MSI-1 siRNA sequences used.

| **siRNA** | **sequence** |
| --- | --- |
| MSI-1 siRNA No 1 | GCUCGACUCCAAAACAAUU |
| MSI-1 siRNA No 2 | GGACGUGAAGCAAUAUUUU |

**Supplementary Table S2.** qPCR TaqMan probe numbers. All details can be found on the manufacturer’s website (Thermo Fisher Scientific).

| **Gene** | **Manufacturer’s ID** |
| --- | --- |
| 18S | hs99999901_s1 |
| Musashi-1 | hs00159291_M1 |
| Notch-1 | hs00413187_M1 |
| Notch-3 | hs01128541_M1 |
| Tdgf1 | hs02339497_g1 |
| Nestin | hs04187831_g1 |
| Nanog | hs02387400_g1 |

**Supplementary Table S3.** MSI-1 siRNA sequences used.

| **Gene** | **Primary antibody** | **Secondary antibody** |
| --- | --- | --- |
| P21 | Rabbit monoclonal IgG  Cell Signalling Technology, Danvers, USA | Goat Anti-Rabbit IgG, H & L Chain Specific Peroxidase Conjugate  Calbiochem, Merck KGaA, Darmstadt |
| Tubulin | Mouse monoclonal IgG  Sigma-Aldrich, St. Louis, USA | Goat Anti-Mouse IgG, H&L Chain Specific Peroxidase Conjugate  Calbiochem, Merck KGaA, Darmstadt |
| MSI-1 | Mouse monoclinal IgG  Santa Cruz, Dallas, USA | Goat Anti-Mouse IgG, HRP conjugated  R&D Systems, Minneapolis, USA |


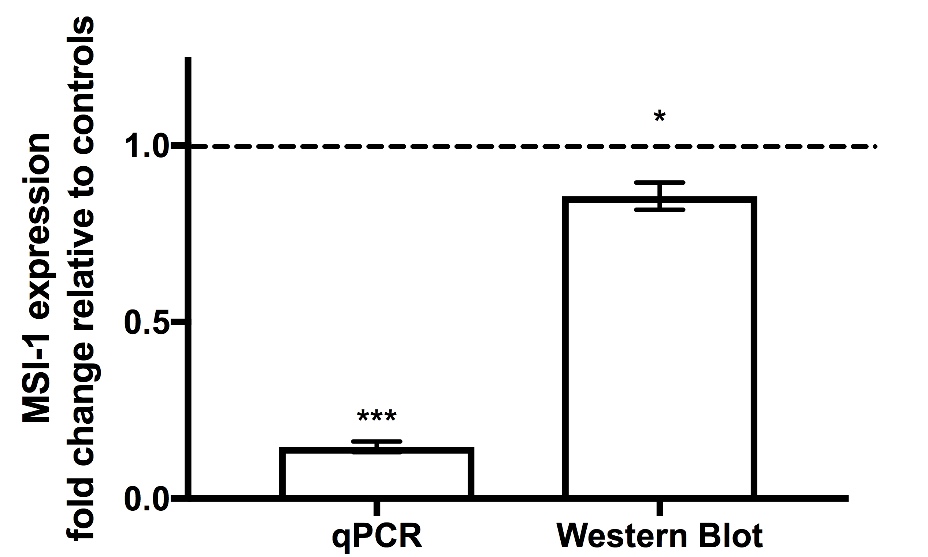


**Supplementary Fig. S1**. MSI-1 expression is downregulated after MSI-1 siRNA transfection, compared to controls. Quantitative polymerase chain reactions (qPCRs) were used for mRNA expression analyses, western blot was used for protein expression analyses. Cells were transfected with a control siRNA and MSI-1siRNA, respectively. Then, measurements were performed as detailed in the Methods section (at least n = 3, * p<0.05, *** p <0.001, error bars indicate s.e.m.).


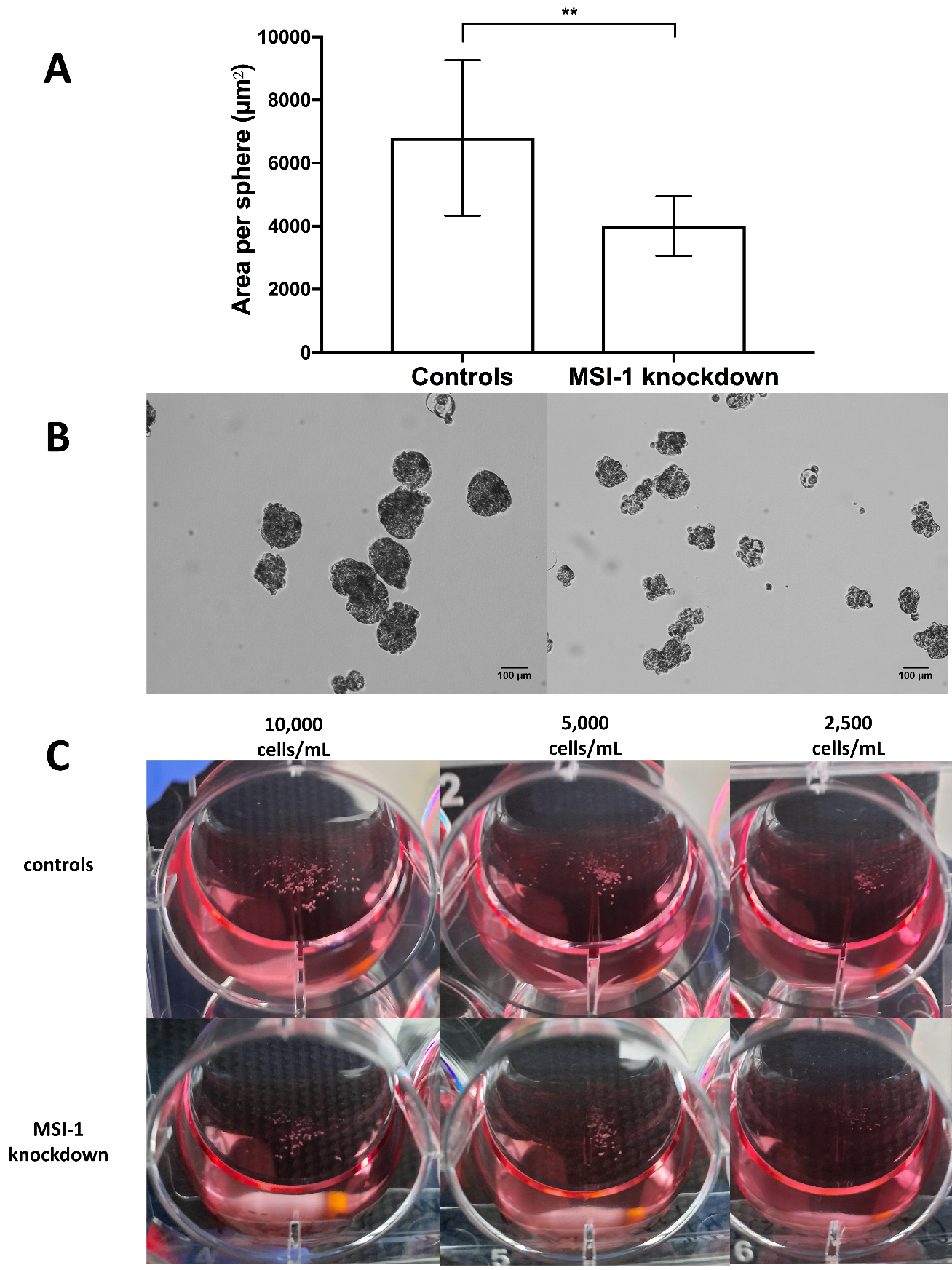


**Supplementary Fig. S2**. Mammosphere formation after MSI-1 knockdown, compared to controls. Equal numbers of cells for controls and after MSI-1 knockdown were seeded in stem cell medium. After 7 days, area per sphere was measured and compared between controls and knockdown cells (**A**). Representative images for controls (left) and knockdown cells (right) in can be found in panel **B**. Macroscopically, fewer spheres were visible as well (**C**). Experiments were performed as detailed in the methods section (at least n = 3, ** p <0.01, error bars indicate s.e.m.).


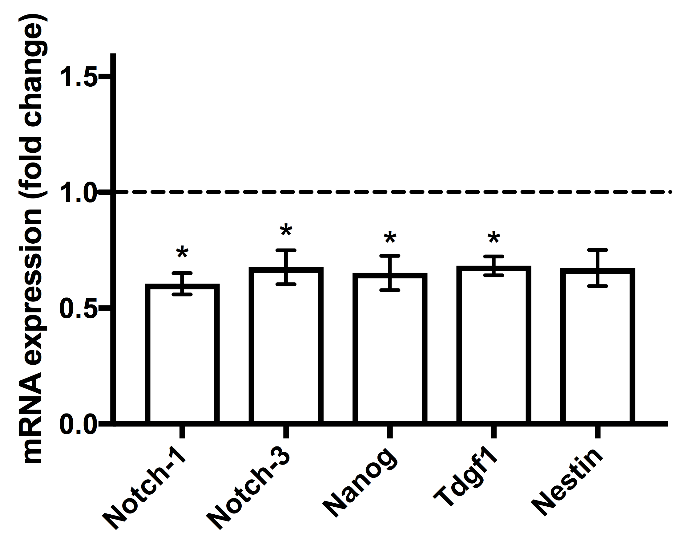


**Supplementary Fig. S3.** Expression of stem cell markers in MCF-7 cells after MSI-1 knockdown, compared to controls. Cells were transfected with a control siRNA and MSI-1 siRNA, respectively. Then, qPCR measurements were performed as detailed in the Methods section (at least n = 3, * p<0.05, error bars indicate s.e.m.).


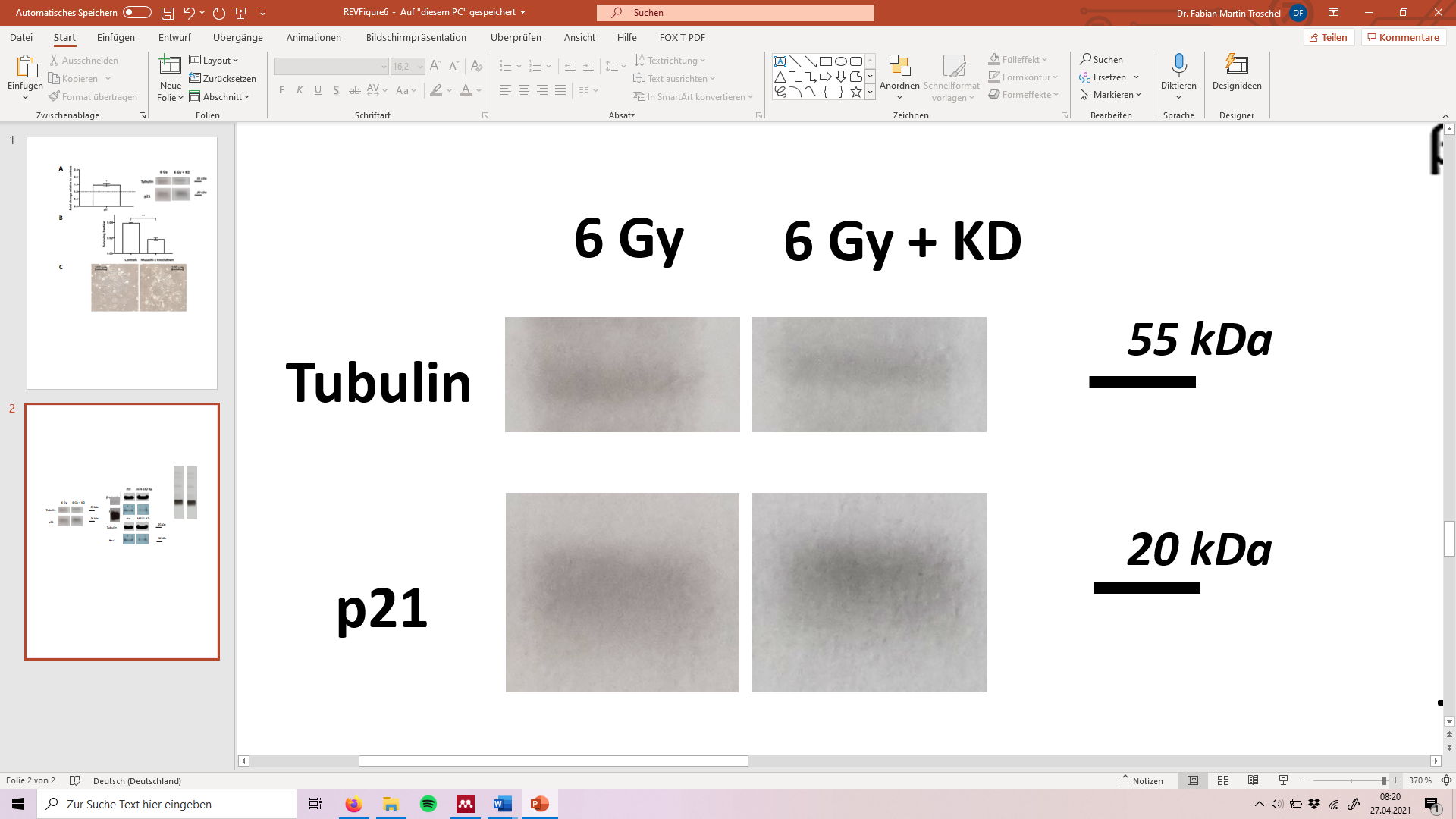


**Supplementary Fig. S4.** Representative blot: p21 is stronger expressed in MSI-1 knockdown cells after 6 Gy irradiation compared to control siRNA transfected cells after 6 Gy.


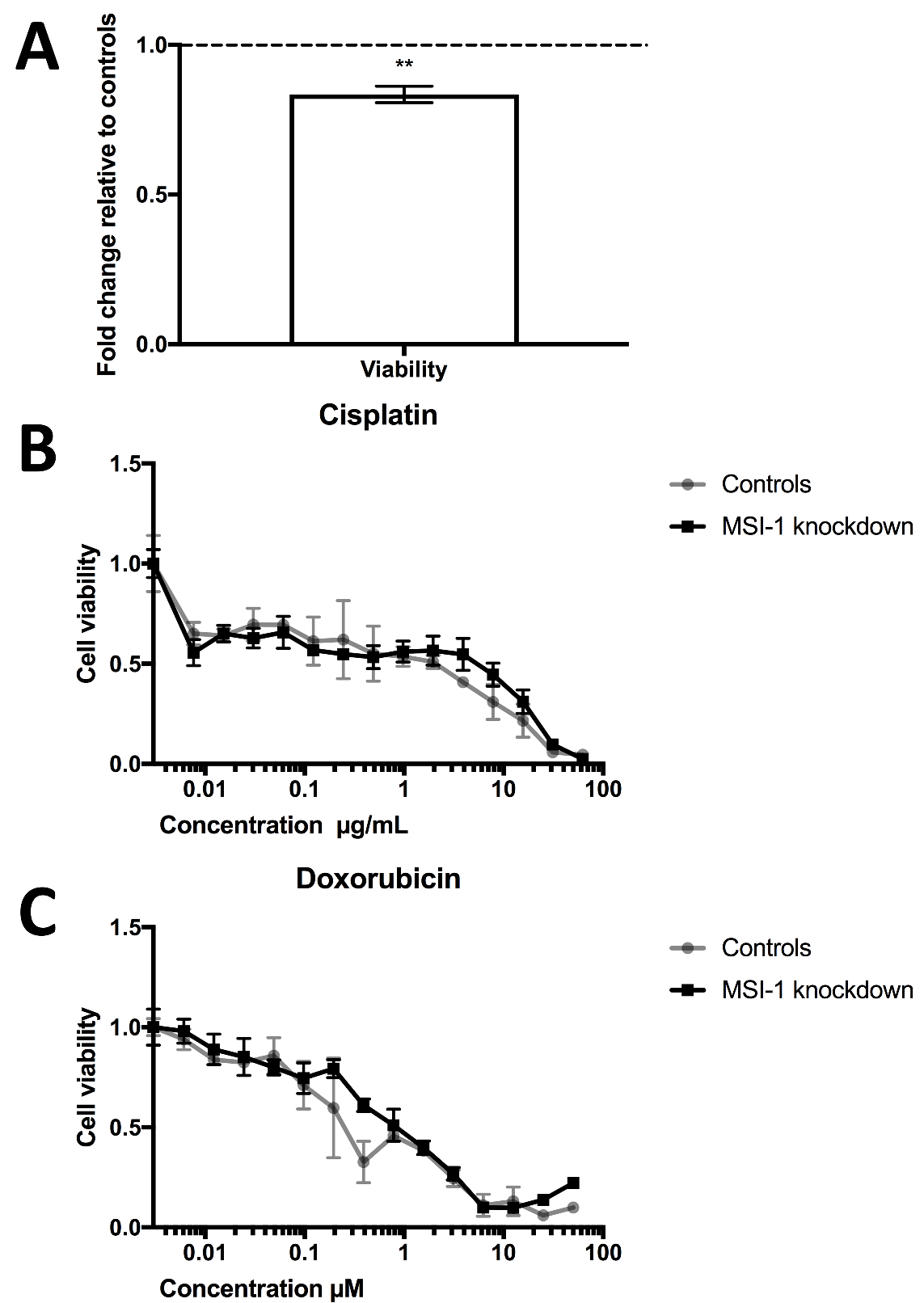


**Supplementary Fig. S5.** Cell viability after MSI-1 knockdown and chemotherapy addition. A: Cell viability is decreased in MSI-1 knockdown cells compared to controls before adding chemotherapy (at least n = 3, ** p<0.01, error bars indicate s.e.m.).. B & C: No additional chemosensitization is seen in MSI-1 knockdown cells after addition of Cisplatin and Doxorubicin.
